# Partial Immobility of the Orbicularis Oris

**Published:** 1849-07

**Authors:** 


*Partial Immobility of the Orbicularis Oris.*—A young lady, about seventeen or eighteen years of age, applied to us, a short time since, for a substitute for the four upper incisores, which she had lost some twelve months before, in consequence of having been severely salivated while affected with remittent fever. Upon examination of her mouth, we discovered that the loss of the teeth in question was not the only inconvenience she had sustained from the salivation. The sphincter muscle of the mouth had been rendered, in consequence of preternatural adhesions of the mucous membrane, in a great degree, immovable. She could close her lips, but the greatest extent to which her mouth could be opened, would barely admit of the introduction of a fifty cent piece. We suggested to her that this inconvenience might be remedied by a simple surgical operation, but were unable to prevail upon her to submit to it.

It was impossible, under the circumstances, to introduce a wax-holder, of the common form, of any size, into her mouth, for the purpose of obtaining an impression. We did, however, ultimately succeed in obtaining an imperfect one of the portion of the alveolar ridge between the cuspidati, and by the aid of which we finally adapted a gold plate, that served as a base for four artificial teeth. The clasps for the retention of the piece in the mouth were adapted to the second bicuspides, after being soldered to the plate.—*Balt. Ed.*

